# Single-lead Thigh ECG Dataset (tOLIet) with Analysis of BMI Effects on Cardiac Signal Quality

**DOI:** 10.1038/s41597-026-06713-6

**Published:** 2026-03-12

**Authors:** Aline Santos Silva, Miguel Velhote Correia, Sérgio Matoso Laranjo, Helena Fonseca, Andreia Cristina Gonçalves da Costa, Hugo Plácido da Silva

**Affiliations:** 1 INESC TEC, Faculdade de Engenharia, Universidade do Porto. R. Dr. Roberto Frias., 4200-465 Porto, Portugal; 2 OLI Sistemas e Sanitários. Travessa do Milão., 3800-314 Aveiro, Portugal; 3 IT - Instituto de Telecomunicações, IST - Instituto Superior Técnico, Universidade de Lisboa. Av. Rovisco Pais - Torre Norte - Piso 10., 1049-001 Lisboa, Portugal; 4 Paediatric Cardiology at the Central Lisbon Hospital and University Centre, Alameda Santo António dos Capuchos, 1169-050 Lisboa, Portugal

**Keywords:** Quality of life, Medical research

## Abstract

In previous work, we introduced an ‘invisible’ ECG system with electrodes integrated into a toilet seat, capturing signals from the thighs. Here, we present the *tOLIet* dataset with single-lead thigh ECGs to advance cardiovascular assessment using this novel approach. The dataset includes 149 records from 86 individuals (50 females, 36 males; mean age 31.73 ± 13.11 years; weight 66.89 ± 10.70 kg; height 166.82 ± 6.07 cm). Participants were recruited via the Centro Hospitalar Universitário de Lisboa Central (CHULC). Each recording features four differential signals from toilet-seat electrodes alongside reference data from a hospital-grade 12-lead ECG. Beyond signal collection and quality evaluation, we conducted a gender-specific analysis comparing valid signal percentages relative to Body Mass Index (BMI). This analysis explores anatomical or physiological factors affecting thigh-based ECG acquisition, guiding system design and customization to enhance signal reliability across populations.

## Background & Summary

Over the last century, the global epidemiological landscape has undergone a substantial transformation. While infectious diseases were the leading cause of mortality in the early 20th century, Chronic Non-communicable Diseases (NCDs) have now become the primary contributors to global death rates^1^. CardioVascular Diseases (CVDs) represent the most prevalent group among NCDs, accounting for more than 30% of all deaths worldwide^2^. Early detection and continuous monitoring of cardiac function, therefore, remain essential for effective management and prevention^3,4^.

ElectroCardioGraphy (ECG) is the gold standard for cardiac assessment, traditionally relying on 12-lead clinical recordings that require trained personnel and controlled environments. To extend monitoring beyond clinical settings, recent years have witnessed a rapid expansion of non-invasive and unobtrusive ECG acquisition technologies. Wearable devices-such as smartwatches, chest straps, adhesive patches, rings, and textile-based sensors-offer convenient long-term monitoring but depend heavily on user compliance, require periodic charging, and are frequently affected by motion artifacts or inconsistent skin contact^5–7^.

Beyond wearables, several researchers have explored ambient or device-integrated approaches for passive ECG acquisition. These include ECG systems integrated into chairs, beds, automobile steering wheels, armrests, and clothing^4,8,9^. Although such solutions reduce user burden, many require specific postures, deliberate contact, or stationary use, limiting their applicability for frequent and fully passive recordings.

In this context, ECG acquisition through toilet seats has emerged as a promising non-contact or minimally obtrusive alternative^10,11^. Toilets are used regularly, typically under consistent environmental conditions, and provide stable thigh-to-electrode contact. These characteristics make them suitable for routine monitoring and for capturing longitudinal cardiovascular trends that may not be detectable during short-term clinical assessments. It is important to note that, although the ECG obtained from the thighs does not reproduce the full diagnostic capability of a 12-lead system, previous studies have shown that reduced-lead or limb-derived ECGs can reliably capture heart rate, rhythm disturbances, conduction intervals, and repolarization abnormalities-parameters of substantial clinical value^12,13^. Thus, reducing the number of leads represents not only convenience but also a viable compromise that enables more frequent, long-term monitoring capable of complementing conventional diagnostics.

Building on these principles, we present a single-center retrospective study evaluating the feasibility of acquiring ECG from four pairs of toilet-seat-embedded electrodes with different textures and positions. The study aims to characterize signal quality, compare valid-signal yield across electrode pairs, and assess physiological variability in acquisition performance. Particular attention is given to the influence of Body Mass Index (BMI) and gender, as anatomical factors may significantly affect electrode-skin contact and, consequently, ECG morphology and signal fidelity^14–16^. By systematically analyzing these factors, the dataset produced here provides practical insights for the development of future unobtrusive cardiovascular monitoring platforms.

## Methods

### Ethics Statement

This study was reviewed and approved by the Ethics Committee of the Centro Hospitalar Universitário de Lisboa Central (CHULC), Portugal, under approval number INV 496. An independent evaluation by the institution’s Data Protection Officer concluded that the potential risks to participants’ rights and freedoms were minimal. This conclusion was based on multiple criteria, including the voluntary and informed nature of participant enrollment and the robust anonymization procedures applied to the data. Each participant’s physiological signals were labeled using unique study-specific codes that bear no link to personal identifiers, thereby ensuring complete anonymity. Before participation, all individuals received a clear explanation of the study’s objectives, data acquisition procedures, and confidentiality safeguards. Participation was entirely voluntary, and no monetary compensation was offered.

### Participants

A total of 86 individuals, all of Portuguese nationality, voluntarily participated in this study. The group included 50 females and 36 males, with a mean age of 31.73 ± 13.11 years, a mean body weight of 66.89 ± 10.70 kg, and a mean height of 166.82 ± 6.07 cm. The BMI was estimated for each participant using the standard formula (weight in kilograms divided by height in meters squared), resulting in a mean BMI of approximately 23.95 ± 3.95 kg/m^2^. According to the World Health Organization classification, this average value falls within the ‘normal weight’ category (18.5-24.9 kg/m^2^), although some individual variation was present across the cohort.

Participants were recruited through direct contact with the principal investigator at CHULC, and all clinical appointments and data collections took place there. While a few participants self-reported possible cardiac conditions, no official clinical diagnoses were available for confirmation. The cohort was intentionally diverse in terms of age and body composition to enhance the generalizability of the results. To the best of our knowledge, this is the first publicly available dataset consisting of thigh-based electrocardiographic recordings.

### Experimental Setup and Protocol

ECG signals were collected using a custom-developed system integrated into a standard toilet seat installed in a domestic bathroom, ensuring ecological validity. The system consists of four sensor modules, each containing a pair of dry electrodes designed to record a single-lead ECG signal (ECG_EXP). The four electrode pairs differ in surface texture (flat, sinusoidal, pyramidal, and trapezoidal) and are positioned in different lateral regions of the seat to evaluate how texture and anatomical contact affect signal quality. A detailed description of the mechanical and electrical design of the sensors is provided in^17^.

**Fig. 1:**
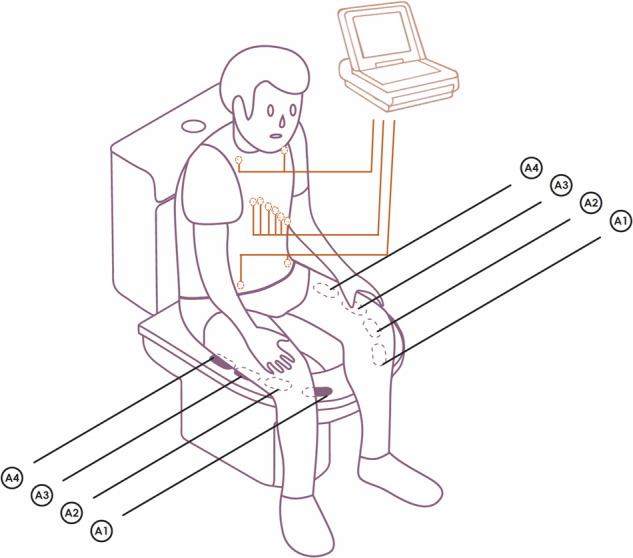
Experimental setup showing the GE HealthCare MAC800 system (ECG_REF) and the toilet-seat-based acquisition system (ECG_EXP), which uses dry electrodes placed on the thighs. Adapted from^17^.

ECG signals were transmitted wirelessly via Bluetooth to a laptop running OpenSignals (r)evolution, which managed real-time visualization and data storage. In selected participants, simultaneous clinical-quality recordings were obtained using a GE Healthcare MAC800 12-lead ECG system (ECG_REF) to serve as a reference standard. The complete experimental configuration is shown in Fig. 1. In these cases, the reference electrodes were positioned on the participant’s torso, and once seated and stabilized, the reference acquisition was performed, limited to 10-second recordings.

Signal acquisition was implemented using the BITalino platform (PLUX S.A., Lisbon, Portugal), previously validated for biosignal research^17,18^. The analog front-end consisted of differential amplification with a gain of 11,000, a hardware bandpass filter with cutoff frequencies of 0.5-40 Hz, 10-bit ADC resolution, and a sampling rate of 1,000 Hz per channel.

Participants used the toilet naturally, without posture or time restrictions. Each recording captured the entire interval from the moment the participant sat down until standing up, resulting in recordings of variable duration, typically up to five minutes. This variability reflects the real-world nature of the study and explains the inconsistencies observed in file lengths. No truncation or artificial segmentation was applied to the raw signals stored in the dataset.

After acquisition, all recordings were manually reviewed to ensure that each session contained at least 10 seconds of valid ECG for each electrode. Segments affected by motion artifacts or intermittent contact loss were not removed from the provided files; instead, the full raw signal is preserved to allow transparency, reproducibility, and secondary analyses by other researchers.

All data processing was performed in Python 3.11.7. For signal preprocessing–including digital filtering and beat segmentation–the BioSPPy package (v2.2.3) was used. The bandpass filter is a linear-phase Finite Impulse Response (FIR) filter with an effective order of 1,500 taps, implemented via direct convolution. The relatively high order ensures smooth frequency response and minimal distortion of the QRS complex.

A 0.5 Hz high-pass cutoff was selected in accordance with ANSI/AAMI/IEC 60601 standards for diagnostic ECG acquisition. The primary objective is the reliable extraction of cardiac timing and conduction features, including heart rate, PR interval, PR segment, QRS duration, ST segment, and QT interval. HRV analysis relies mainly on R-peak timing and is minimally affected by slow baseline fluctuations. By attenuating low-frequency drifts caused by motion or posture–common in real-world toilet use–the 0.5 Hz high-pass filter improves R-peak detectability and reduces false detections without compromising the accuracy of key ECG intervals. HRV metrics and temporal ECG features extracted from the filtered signals were confirmed to be consistent with reference measurements obtained from the GE MAC800 system.

To ensure full reproducibility, all raw signals, processing scripts, and derived data are included in the dataset accompanying this article. Unless otherwise specified, results are reported as mean  ± standard deviation across participants.

## Data Records

**Fig. 2:**
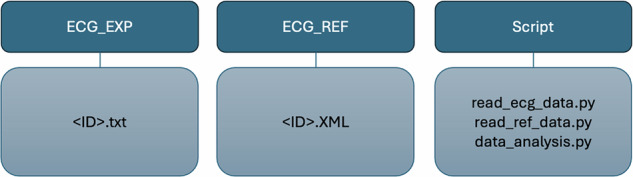
Diagram of the dataset structure.

The dataset was organized to contain the raw data and all the code used to process and analyze it. To facilitate the reproducibility and transparency of the research, the graphics and tables derived during the research process are also shared. Figure 2 shows an overview of the dataset structure.

**Fig. 3:**
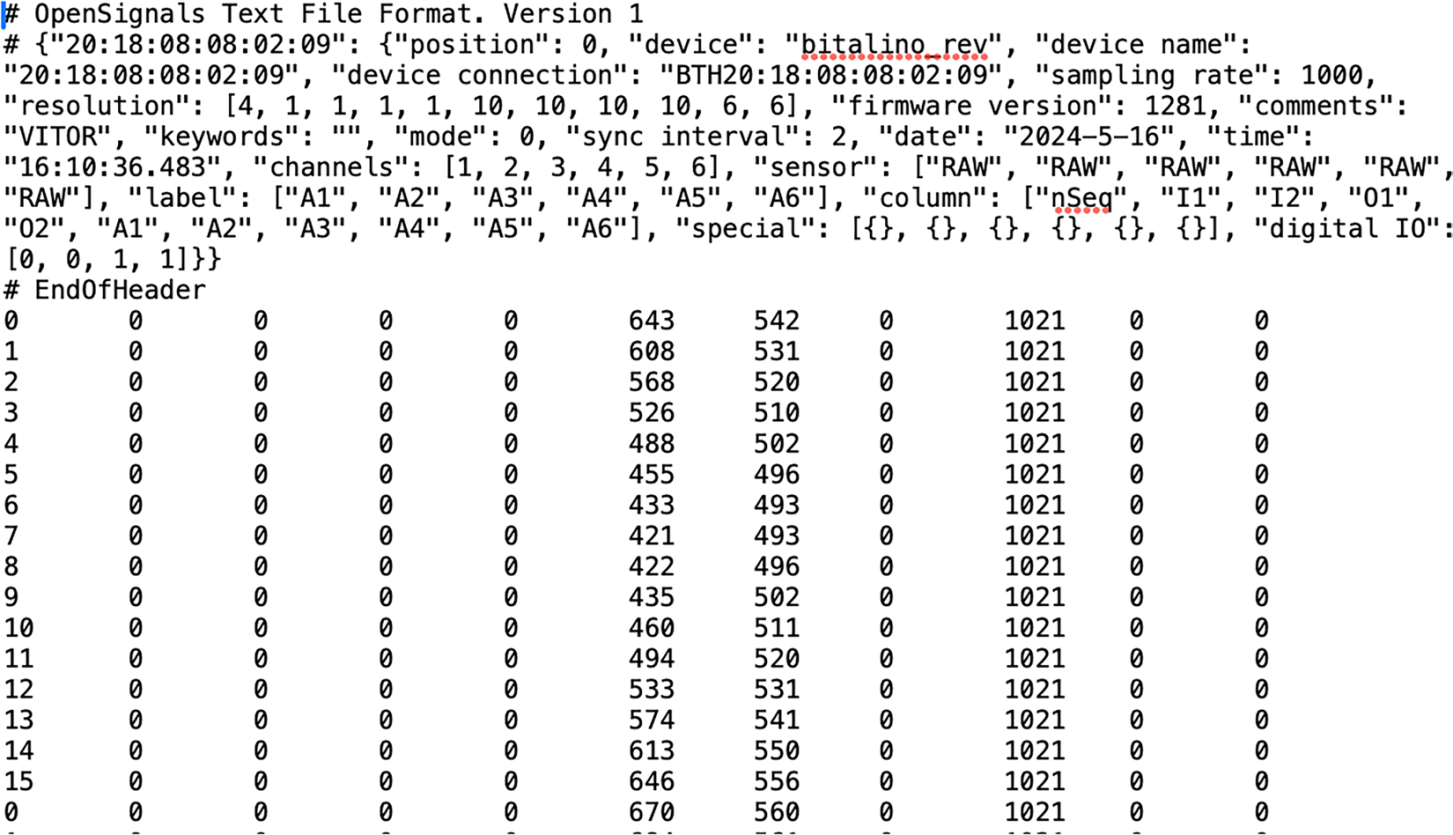
Example: the TXT file in the ECG_EXP folder. The description of the header structure can be found in^22,23^.

The dataset contains three main folders, described as follows:

ECG_EXP– Contains the files extracted from the toilet seat. Each TXT file is structured as follows (Fig. 3):

**nSeq** (*Column 1*): Numerical sequence used to verify if there has been signal loss.

*Column 2 & 3*: BITalino digital inputs I1 & I2 (not used).

*Column 4 & 5*: BITalino digital outputs O1 & O2 (not used).

**A1** (*Column 6*): ECG signal extracted from sensor A1 (with flat electrode texture).

**A2** (*Column 7*): ECG signal extracted from sensor A2 (with sinusoidal electrode texture).

**A3** (*Column 8*): ECG signal extracted from sensor A3 (with pyramidal electrode texture).

**A4** (*Column 9*): ECG signal extracted from sensor A4 (with trapezoidal electrode texture).

*Column 10 & 11*: BITalino analog inputs A5 & A6 (not used).

ECG_REF– Contains the files extracted from the reference system; characteristics can be extracted from these files using the *read_ref_data.py* function, which will return:

**id** - Subject identification code.

**acquisitionDateTime** - The date and time of the acquisition.

**name_dev** - The identification of each lead acquired (e.g. I, II, III, AVR, AVL, AVF, V1, V2, V3, V4, V5, V6).

**meanTemplate**- Average of the heartbeat models extracted for each lead.

**dev** - Time series signal extracted for each lead over a period of 10s.

**unit**- Unit of the extracted signal (uV).

**Scripts** - Contains three main scripts with codes for extracting signal characteristics, namely:

*read_ecg_data.py* - Reads the raw physiological signals collected with ECG_EXP; the annotations transformed data in separate dictionaries (with matrix data for the session) and annotated data segments.

*read_ref_data.py* - Reads the raw physiological signals collected with ECG_REF; the annotations transform the data into separate dictionaries containing matrix-structured session data and annotated segments. The script read_ref_data.py is a custom XML parser developed specifically for ECG reference files exported by the GE MAC800 recorder (Sapphire/DCAR format). The function read_ref() reads the XML structure using fixed paths that correspond to the GE MAC800 export schema. It extracts: (i) the acquisition date and time from testInfo/acquisitionDateTime; (ii) the name of each of the 12 leads; (iii) the median beat template for each lead from the node var/medianTemplate/ ecgWaveformMXG; and (iv) the corresponding 10-second raw waveform from wav/ecgWaveformMXG. In both cases, the numerical samples are parsed directly from space-separated strings contained in the XML. The script assumes the presence of exactly 12 leads in a fixed order and that all waveform entries follow the GE MAC800 formatting. Because these node names, hierarchical paths, and sample formats are specific to the GE MAC800, users wishing to apply this script to ECG recorders from other manufacturers must manually adapt the code by modifying the XML paths from which the acquisition metadata, lead identifiers, median templates, and waveform values are extracted. This requires inspecting the alternative recorder’s XML structure and replacing the corresponding nodes inside the loops that read auxT (templates) and auxD (waveforms). All other processing steps remain unchanged, and no additional assumptions beyond the GE MAC800 XML structure are embedded in the script.

**data_analysis.py** - Script used to obtain the graphs and tables presented in the technical validation section.

In addition to these main folders, the spreadsheet **dataSet.csv** contains information about the study population, in particular: ID, Age, Weight, Height and Gender.

## Technical Validation

Tables 1 and 2 contains information about file ***dataSet.csv*** and also shows the percentage of valid signals extracted from each electrode pair for each subject. The file ***dataSet.csv*** also has a field for observations in which annotations were made for the subjects with self-reported cardiac conditions (Table 3).

**Table 1:** Descriptive analysis of the characteristics of the participants, the ECG signals collected and the percentage of valid signal for each pair of electrodes extracted from the EXP ECG (Part I).

ID	Age (years)	Weight (kg)	Height (cm)	Gender	% SV (A1)	% SV (A2)	% SV (A3)	% SV (A4)
1	40	77	170	Male	60	70	0	70
2	41	78	171	Male	100	100	0	0
3	38	79	173	Male	100	100	0	0
4	36	81	171	Male	90	90	0	90
5	36	60	171	Male	70	60	0	20
6	27	61	162	Female	100	0	0	0
7	27	61	162	Female	56	100	0	0
8	27	61	162	Female	0	100	0	0
9	27	61	162	Female	90	0	0	0
10	82	82	170	Male	55	0	0	0
11	64	70	167	Male	0	100	0	0
12	34	64	159	Female	100	0	96	0
12_1	34	64	159	Female	100	100	0	100
13	27	51	161	Female	100	0	70	0
13_1	27	51	161	Female	100	100	0	100
14	32	60	169	Female	100	0	0	0
14_1	32	60	169	Female	100	100	0	0
15	35	51	174	Male	100	0	0	0
15_1	35	51	174	Male	100	100	0	0
16	25	69	160	Female	100	0	0	0
16_1	25	69	160	Female	100	100	0	0
17	23	81	172	Male	100	0	0	0
17_1	23	81	172	Male	100	100	0	0
18	31	68	167	Female	100	0	0	0
18_1	31	68	167	Female	100	100	0	0
19	24	72	171	Male	100	0	0	0
19_1	24	72	171	Male	100	100	0	0
20	26	67	164	Female	100	0	0	0
20_1	26	67	164	Female	100	100	0	0
21	36	63	162	Female	100	0	0	0
21_1	36	63	162	Female	100	100	0	0
22	28	78	172	Male	100	0	0	0
22_1	28	78	172	Male	100	100	0	0
23	26	49	172	Male	100	0	0	0
23_1	26	49	172	Male	100	100	0	0
24	30	71	163	Female	100	0	0	0
24_1	30	71	163	Female	100	100	0	0
25	21	77	174	Male	100	0	0	0
25_1	21	77	174	Male	98	96	0	0
26	34	61	159	Female	100	100	0	0
26_1	34	61	159	Female	100	100	0	0
27	26	53	175	Male	98	0	0	0
27_1	26	53	175	Male	100	100	0	0
28	22	67	166	Female	100	100	0	0
28_1	22	67	166	Female	100	0	0	30
29	19	50	159	Female	100	100	0	0
29_1	19	50	159	Female	55	45	0	0
30	32	50	171	Male	100	100	0	0
30_1	32	50	171	Male	55	45	0	0
31	31	57	169	Male	100	100	0	0
31_1	31	57	169	Male	93	98	0	0
32	23	74	167	Female	100	100	0	0
32_1	23	74	167	Female	65	20	0	40
33	36	75	167	Female	100	100	0	0
33_1	36	75	167	Female	90	85	0	85
34	32	80	163	Female	100	100	0	0
34_1	32	80	163	Female	100	100	0	100
35	26	52	171	Male	100	96	0	0
35_1	26	52	171	Male	98	98	0	98
36	32	59	168	Female	98	98	0	0
36_1	32	59	168	Female	100	100	0	100
37	23	63	168	Female	100	100	0	0
37_1	23	63	168	Female	100	100	0	100
38	22	76	171	Male	100	100	0	0
38_1	22	76	171	Male	80	80	0	70
39	36	60	162	Female	100	100	0	0
39_1	36	60	162	Female	100	65	0	0
40	28	64	168	Female	100	100	0	0
40_1	28	64	168	Female	100	0	0	0
41	33	62	167	Female	100	100	0	0
41_1	33	62	167	Female	100	0	0	0
42	30	77	161	Female	100	100	0	0
42_1	30	77	161	Female	100	0	0	0

**SV** — Percentage of valid ECG signal (without saturation) acquired by electrode pair A1-A4.

**Table 2:** Descriptive analysis of the characteristics of the participants, the ECG signals collected and the percentage of valid signal for each pair of electrodes extracted from the EXP ECG (Part II).

ID	Age (years)	Weight (kg)	Height (cm)	Gender	% SV (A1)	% SV (A2)	% SV (A3)	% SV (A4)
43	34	62	164	Female	98	98	0	0
43_1	34	62	164	Female	100	0	0	0
44	29	67	175	Male	100	100	0	0
44_1	29	67	175	Male	100	0	0	100
45	32	55	162	Female	100	100	0	0
45_1	32	55	162	Female	100	0	0	100
46	26	75	171	Male	100	100	0	0
46_1	26	75	171	Male	100	0	0	100
47	19	68	162	Female	100	100	0	0
47_1	19	68	162	Female	97	0	0	97
48	23	77	166	Female	100	100	0	0
48_1	23	77	166	Female	96	0	0	100
49	25	56	173	Male	100	100	0	0
49_1	25	56	173	Male	100	100	0	100
50	29	67	173	Male	100	100	0	0
50_1	29	67	173	Male	100	100	0	100
51	21	56	159	Female	100	100	0	0
51_1	21	56	159	Female	100	100	0	100
52	26	63	165	Female	100	100	0	0
52_1	26	63	165	Female	100	100	0	100
53	19	60	175	Male	100	100	0	0
53_1	19	60	175	Male	100	100	0	100
54	30	68	161	Female	100	100	0	0
54_1	30	68	161	Female	100	100	0	100
55	26	71	170	Female	100	100	0	0
55_1	26	71	170	Female	100	100	0	100
56	27	50	162	Female	100	100	0	0
56_1	27	50	162	Female	100	100	0	100
57	27	56	171	Male	100	100	0	0
57_1	27	56	171	Male	70	0	0	0
58	19	59	166	Female	100	100	0	0
58_1	19	59	166	Female	60	0	0	0
59	26	71	160	Female	100	100	0	0
59_1	26	71	160	Female	100	100	0	100
60	24	69	165	Female	100	100	0	0
60_1	24	69	165	Female	70	0	0	0
61	27	70	170	Female	100	100	0	0
61_1	27	70	170	Female	100	85	0	0
62	35	67	171	Male	100	100	0	0
62_1	35	67	171	Male	100	100	0	0
63	30	61	171	Male	92	92	0	92
63_1	30	61	171	Male	100	100	0	80
63_2	30	61	171	Male	100	100		100
64	29	53	174	Male	100	100	0	100
64_1	29	53	174	Male	100	100	0	100
64_2	29	53	174	Male	100	100	0	100
65	21	79	171	Male	100	100	0	100
65_1	21	79	171	Male	100	100	0	100
65_2	21	79	171	Male	100	100	0	0
66	32	80	161	Female	100	100	0	100
66_1	32	80	161	Female	100	100	0	100
66_2	32	80	161	Female	100	100	0	0
67	30	77	161	Female	100	100	0	100
67_1	30	77	161	Female	100	100		100
67_2	30	77	161	Female	0	0	0	100
68	24	80	159	Female	100	100	0	0
68_1	24	80	159	Female	100	100		100
68_2	24	80	159	Female	0	0	0	100
69	25	54	164	Female	0	45	0	20
70	33	75	170	Male	30	100	100	0
71	27	53	160	Female	98	100	70	98
72	24	58	168	Female	80	90	10	0
73	24	61	160	Female	98	96	0	100
74	22	53	163	Female	94	100	0	0
75	23	75	185	Male	0	0	0	57
76	72	69	171	Male	100	80	100	100
77	24	60	171	Male	100	0	0	0
78	18	83	159	Female	0	0	0	100
79	68	56	155	Female	0	55	0	0
80	61	78	176	Male	0	98	0	99
81	68	71	150	Female	0	0	70	75
82	83	76	156	Female	0	100	100	0
83	37	91	180	Male	100	100	0	0
84	38	95	178	Male	70	85	0	85
85	28	61	163	Female	100	100	0	0
86	36	95	167	Male	85	85	0	85

**SV** — Percentage of valid ECG signal (without saturation) acquired by electrode pair A1-A4.

**Table 3:** Summary of the subjects with self-reported clinical history.

ID	Observations
70	Paroxysmal Atrial fibrillation (AF) after ablation
76	Paroxysmal AF + left bundle branch block + cardiomyopathy under study
79	Paroxysmal AF (1 month after ablation)
80	Paroxysmal AF

The difference observed between the electrode pairs is not directly related to the electrode texture, but rather to the position of each electrode pair and to the way the user sits on the toilet, which determines the degree of effective contact with each pair. Additionally, another relevant characteristic of the dataset arises from the influence of users’ body stature on signal acquisition: variations in leg length and overall anthropometry affect how the ECG propagates along the thigh, leading to measurable differences in signal quality depending on the position along the leg. This factor must therefore be considered when evaluating signal consistency and electrode performance.

As previously described, the prototype included four electrode pairs, each with a distinct texture and located in different positions on the toilet seat (Fig. 1). However, as demonstrated in the previously published study^17^, the main factor influencing signal quality is not the shape or texture of the electrode, but the contact geometry between the skin of the thighs and the surface of the seat, which varies according to the user’s posture.

Thus, the percentage of valid signals presented in Tables 1 and 2 mostly reflects differences in contact between each subject and each electrode pair, rather than intrinsic performance differences arising from electrode textures. In other words, the electrode pairs show different values primarily because they do not occupy the same position, and some users establish preferential contact with only one of the lateral regions of the toilet seat.

To further characterize the collected data, we provide a quality assessment of the recorded signals. We began by processing the experimental ECG signals (ECG_EXP), removing saturated portions of the waveform. After artifact removal, the signals were filtered, and a set of standard ECG features was extracted, namely:

**HR**— Heart rate in beats per minute (BPM).

**PR Interval (ms)**— Time interval from the onset of the P wave to the onset of the QRS complex.

**PR Segment (ms)**— The isoelectric interval between the end of the P wave and the beginning of the QRS complex.

**QRS Complex (ms)**— Duration of ventricular depolarization.

**ST Segment (ms)**— Interval between the end of the QRS complex and the onset of the T wave.

**QT Segment (ms)**— Time from the beginning of the QRS complex to the end of the T wave, reflecting the global ventricular action potential duration.

It is important to clarify that the ECG features extracted from the experimental signals (ECG_EXP) were computed using the same physiological principles for wave delineation (P, Q, R, S, T) implemented in our analysis pipeline (data_analysis.py), which relies on the BioSPPy library. These algorithms replicate the standard delineation logic used in clinical ECG processing. Although the exact implementation used for the reference signals (ECG_REF) is part of GE’s proprietary software and therefore confidential, the extraction of ECG_EXP features follows the same conceptual and physiological criteria, ensuring methodological consistency and allowing for a valid comparison between both datasets.

Furthermore, all reported ECG parameter values (HR, PR interval, PR segment, QRS duration, ST segment, and QT interval) were derived from a beat-averaged ECG template computed for each recording, rather than from individual heartbeats. Specifically, a representative template was generated per subject by aligning and averaging consecutive beats, and all morphological features were extracted from this template. This approach provides robust and stable estimates by reducing beat-to-beat variability and noise influence.

**Fig. 4:**
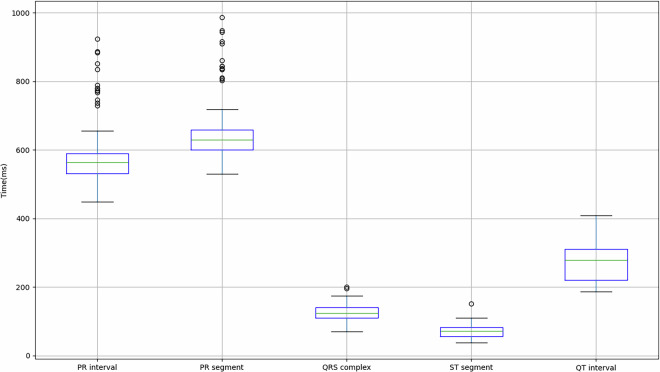
Mean and standard deviation of the study participants’ PR interval, PR segment, QRS complex, ST segment and QT segment.

Figure 4 shows the values obtained for the 149 records in relation to the mean and standard deviation of the PR interval, PR segment, QRS complex, ST segment and QT segment of the study subjects. As previously mentioned, some subjects indicated that they had cardiac conditions (Table 3), so in order to take this into account, Table 4 shows the values obtained for these subjects in comparison with the values obtained for subjects who did not indicate any pathologies.

**Table 4:** Comparison of parameters extracted from the signal in relation to the total average of the subjects for each case study.

ID	HR (BPM)	PR Interval (ms)	PR Segment (ms)	QRS Complex (ms)	ST Segment (ms)	QT Segment (ms)
Others	90.92 ± 10.41	579.57 ± 82.98	646.71 ± 82.34	126.16 ± 22.98	71.32 ± 17.81	273.45 ± 52.78
70	76.75 ± 4.41	612.45 ± 146.15	675.97 ± 137.57	167.53 ± 52.17	70.31 ± 23.63	393.24 ± 54.09
76	78.48 ± 5.55	543.03 ± 195.12	618.66 ± 189.40	174.32 ± 36.12	107.44 ± 99.10	395.48 ± 94.50
79	65.20 ± 0.35	834.50 ± 9.77	915.57 ± 31.03	105.33 ± 10.60	92.72 ± 8.93	362.08 ± 1.89
80	67.29 ± 1.54	789.38 ± 91.87	860.36 ± 86.81	102.88 ± 49.68	98.55 ± 67.76	309.36 ± 120.99

**Fig. 5:**
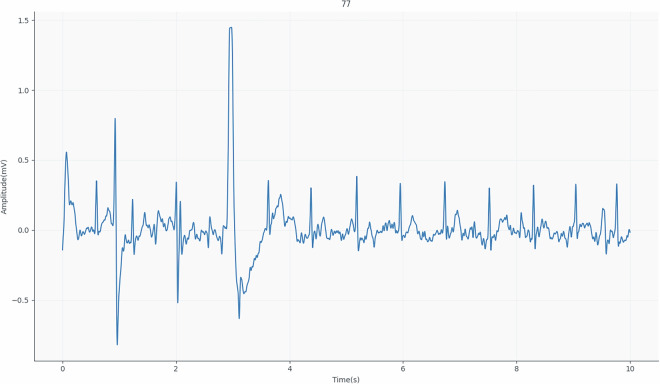
Illustrative ECG_EXP signal extracted from participant 77 during 10 seconds.

**Fig. 6:**
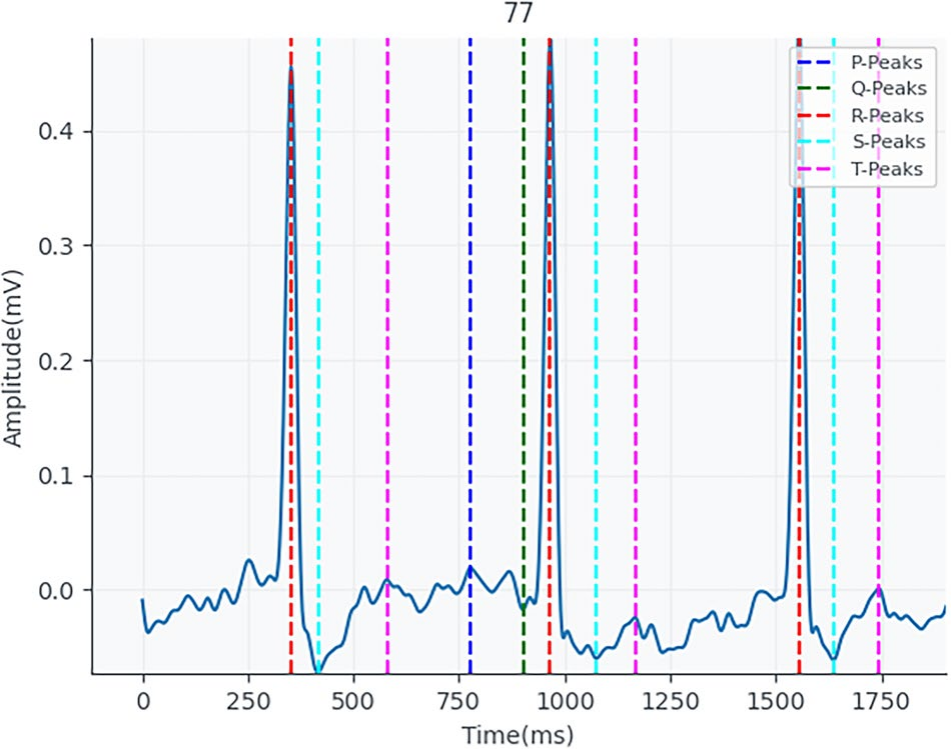
Illustrative ECG_EXP signal extracted from participant 77.

In Fig. 5, we can see the signals extracted by the toilet seat and the position indicators of the extracted complexes. Because the morphology of the thigh ECG signal differs from that of the standard acquisition, it is difficult to identify the P, Q and T complexes. In addition to the waveforms with different visual appearances, the fact that we are dealing with low-amplitude signals makes them prone to some deformation or loss of information. Figure 6 shows the ease with which participant 77’s signal can be deformed and lost. As these are low-amplitude signals, it is easy for the baseline to fluctuate due to factors such as breathing and the participant’s movements during signal collection.

**Fig. 7:**
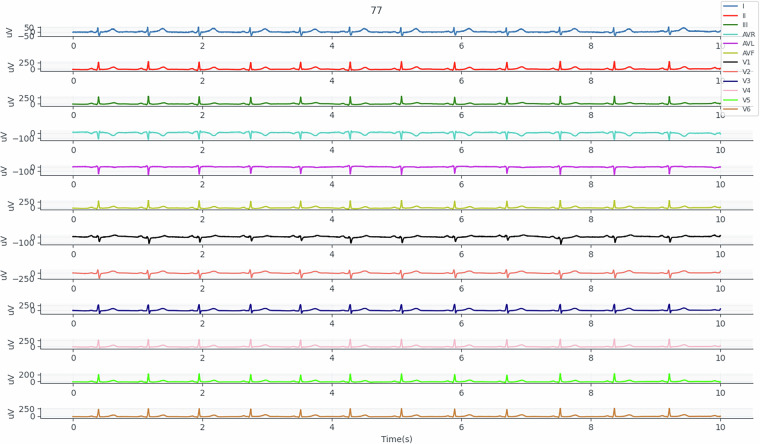
Illustrative ECG_REF signal collected from participant 77.

**Fig. 8:**
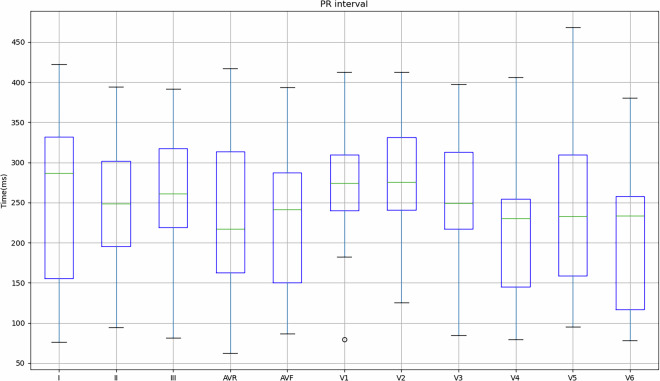
Mean and standard deviation of the PR interval for each lead in relation to the signals obtained on the REF ECG.

**Fig. 9:**
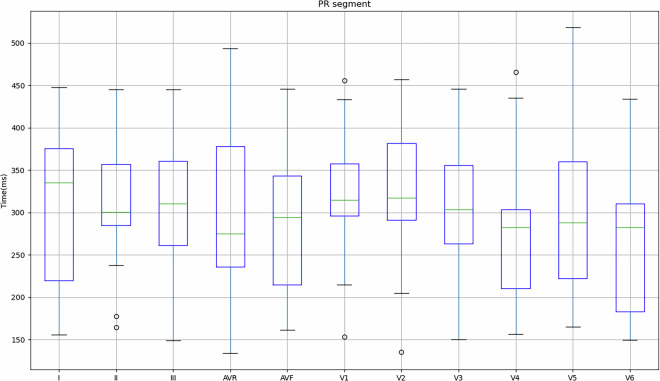
Mean and standard deviation of the PR segment for each lead in relation to the signals obtained on the REF ECG.

**Fig. 10:**
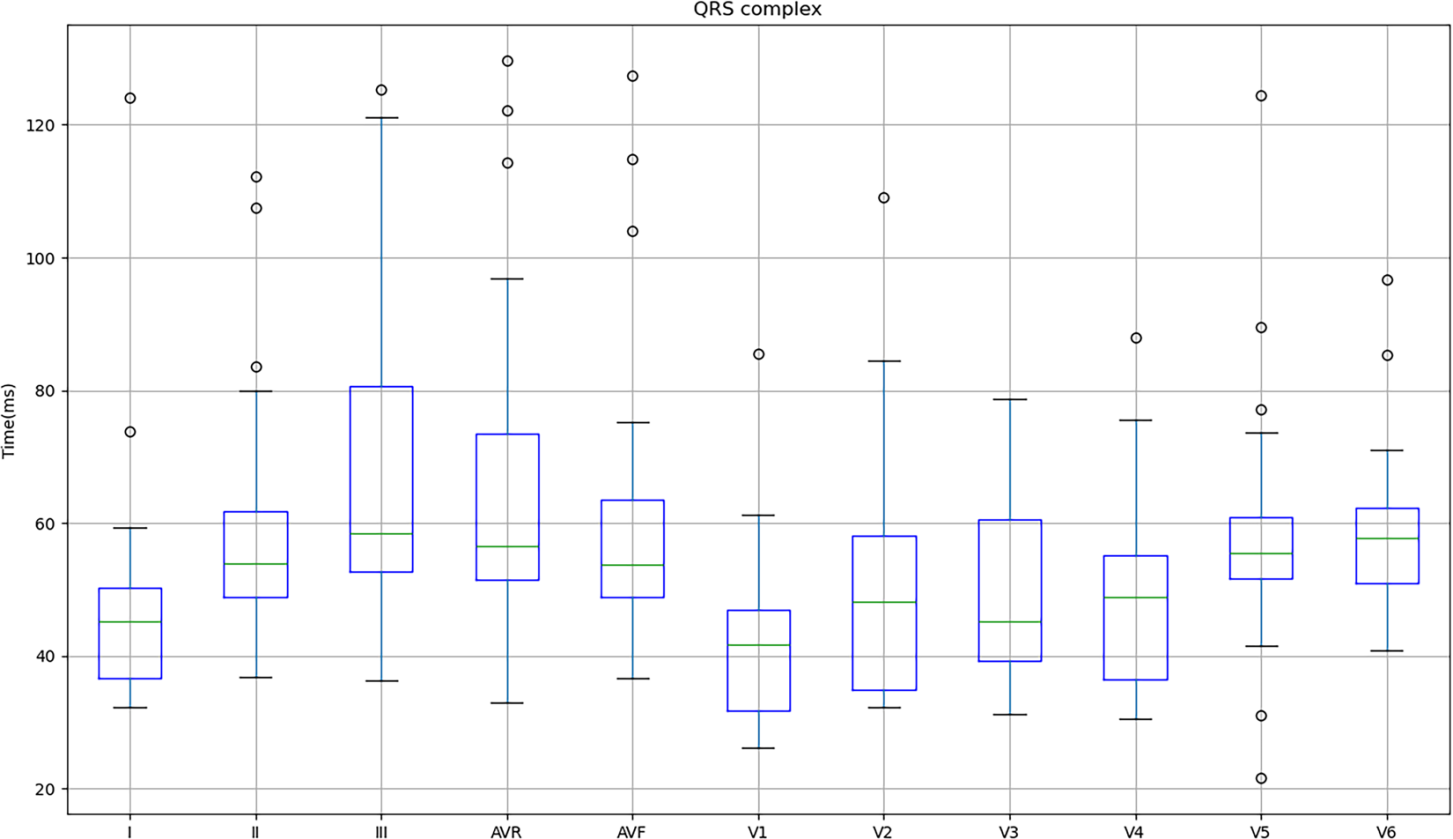
Mean and standard deviation of the QRS complex for each lead in relation to the signals obtained on the REF ECG.

**Fig. 11:**
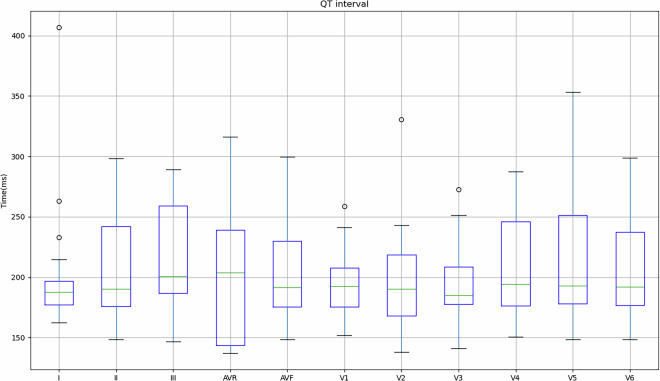
Mean and standard deviation of the QT segment for each lead in relation to the signals obtained on the REF ECG.

**Fig. 12:**
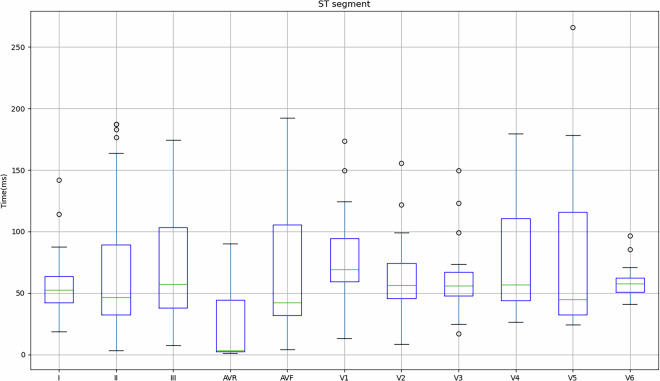
Mean and standard deviation of the ST segment for each lead in relation to the signals obtained on the REF ECG.

In addition to this analysis, we also extracted the signals obtained with the reference system (Fig. 7) and the features of each lead (Figs. 8, 9, 10, 11 and 12). Having access to these signals allows us to explore different metrics, such as the correlation of the 12 leads with the ECG signal obtained by the toilet seat.

**Fig. 13:**
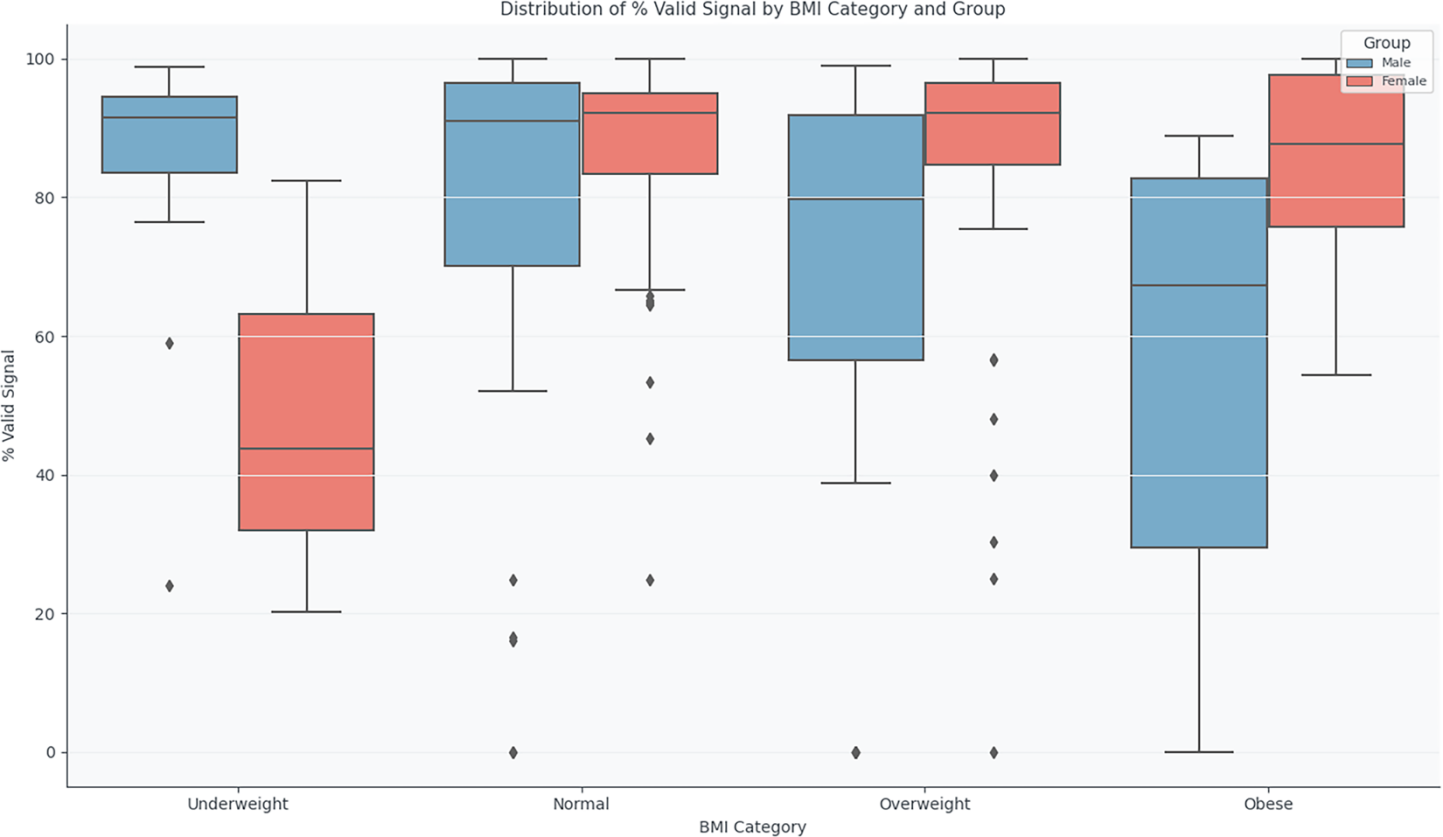
Distribution of % Valid Signal across BMI categories and groups (Male and Female). It is important to note that most male participants did not remove their shirts during the trials. This may have negatively affected the skin-electrode contact, particularly in the overweight and obese categories, contributing to the lower percentage of valid signal observed in these groups.

Figure 13 illustrates the distribution of the percentage of valid ECG signal across different BMI categories, stratified by gender. Female participants consistently demonstrated high and stable signal quality regardless of BMI category. In contrast, male participants, particularly those classified as overweight or obese, exhibited greater variability and a marked decrease in the percentage of valid signal.

It is important to note that during most of the trials, the male participants did not remove their underwear. This probably contributed to poor electrode-skin contact, particularly in individuals with a higher BMI, and may explain the lower signal quality observed in these subgroups. This difference in procedure should be considered when interpreting the results, as it introduces a potential confounding factor in the validity of the signal related to clothing rather than physiological condition.

Despite these trends, the influence of BMI on signal quality should be interpreted with caution, particularly due to imbalances in sample size between BMI categories. Further studies with larger and more evenly distributed samples are needed to robustly quantify these effects and optimise system calibration for populations with different body types.

## Usage Notes

Our dataset comprises ECG recordings obtained through a non-traditional electrode placement. The primary objective is to analyze and characterize signals captured from the thigh region, exploring the feasibility and potential of this novel derivation. This unique data acquisition approach has enabled the collection of a substantial volume of signals, which remain largely unexamined in existing research. Moreover, due to the unconventional nature of the data, it presents an opportunity to apply deep learning techniques that benefit from large datasets to achieve robust performance.

Previous studies from our research group have already investigated various aspects of this system, including optimization of electrode materials^19^ and surface textures^17^ to enhance signal quality, as well as subject identification using single-lead thigh ECG signals^20,21^.

The data acquisition protocol and the associated hardware and software introduce specific constraints, which we outline below. Given the sensitive nature of the measurement site, data collection was conducted in private settings such as bathrooms or hospital rooms, where participants were required to remove lower garments to ensure direct skin-electrode contact. Notably, recordings were made without actual toilet use; the modified toilet seat was placed on a toilet bowl, and subjects remained seated for no longer than five minutes.

Our methodology sought to mimic realistic conditions that could support widespread deployment. Volunteers were not given strict instructions on seating posture aside from maintaining contact between skin and electrodes. However, numerous factors inherent to this usage scenario–such as body hair, application of moisturizing products, or individual body morphology–may affect signal quality. Since these variables can also influence physiological measurements, future research should aim to include such contextual information in data analysis.

## Data Availability

The dataset is available at PhysioNet^21^. The raw data in ‘csv’, ‘txt’ or ‘xml’ format was transformed into dictionaries containing the relevant data in matrices stored in DataFrame format.

## Code availability

To pre-process the physiological data, we used the BioSPPy library (https://github.com/PIA-Group/BioSPPy), which contains modules for filtering the ECG signals, characteristics and data analysis; to analyse the statistical tests, we used the SciPy library (https://github.com/scipy/scipy). For more information on the raw or transformed data, code incompatibilities or other questions, please contact the corresponding author. The processing was carried out in Python 3.11.7, and the necessary code is available in the Scripts folder in PhysioNet^21^ to replicate it easily.
